# Abdominal Aorta Anastomotic False Aneurysm Leading to Double Focal Vertebral Body Erosion

**DOI:** 10.1055/s-0041-1739485

**Published:** 2022-05-31

**Authors:** Umberto G. Rossi, Francesco Petrocelli, Maurizio Cariati

**Affiliations:** 1 Department of Radiological Area, Interventional Radiology Unit, Ente Ospedaliero Galliera Hospital, Mura delle Cappuccine, Genova, Italy; 2 Department of Radiology and Interventional Radiology, Istituto di Ricovero e Cura a Carattere Scientifico San Martino Policlinic University Hospital, Genova, Italy; 3 Department of Diagnostic and Therapeutic Advanced Technology, Diagnostic and Interventional Radiology Unit, Azienda Socio Sanitaria Territoriale Santi Paolo and Carlo Hospital, Milano, Italy

**Keywords:** aorta, false aneurysm, vertebral body, erosion, imaging

## Abstract

Anastomotic aortic false aneurysm with consequent erosion of vertebral bodies is a very rare event that needs prompt treatment. We report the case of a 71-year-old man with an aortobifemoral graft that was complicated by an uninfected proximal anastomotic pseudoaneurysm with double focal vertebral body erosion.

A 77-year-old male patient underwent open repair with an aortobifemoral graft for abdominal aortic aneurysm (AAA). Five years later on imaging follow-up, abdominal contrast medium-enhanced multidetector computed tomography (MDCT; Fig. 1 ) with axial ( Fig. 1A ), sagittal, and coronal multiplanar reconstruction ( Fig. 1B, C ) and coronal volume rendering technique reconstruction ( Fig. 1D ) demonstrated a 15.6 × 4.1 cm thrombosed pseudoaneurysm (shown with * in Fig.1 ) at the level of the proximal abdominal aorta anastomosis, with a vertical course extending from the diaphragmatic dome to the third lumbar vertebra, associated with focal erosion of the second and third lumbar vertebral bodies (arrowheads), with well-delimited bone sclerotic margins. No MDCT signs of infection were noted. Blood cultures were negative.

**Fig. 1:**
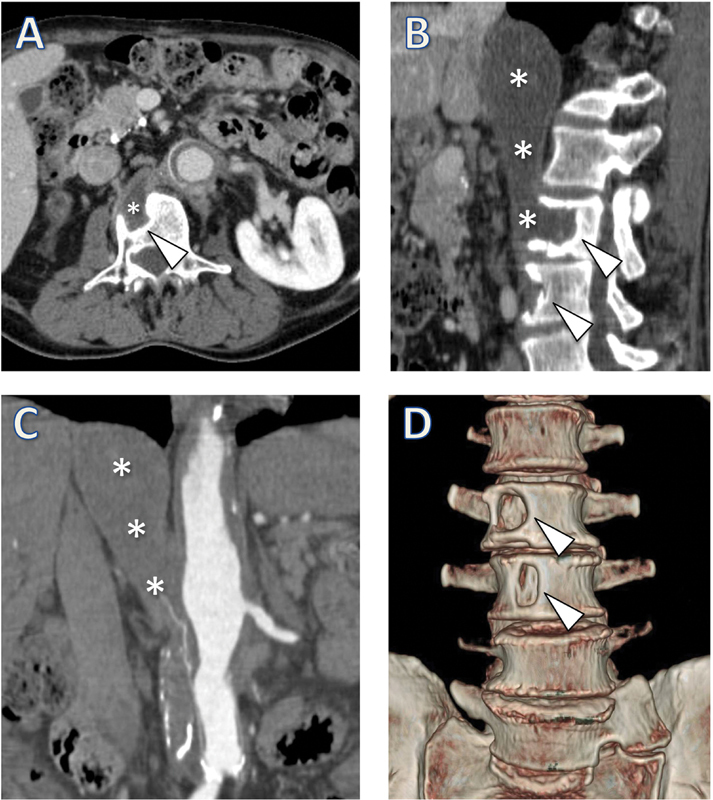
Imaging studies of the reported case. ( **A** ) Axial view. ( **B** ) Sagittal view. ( **C** ) Coronal view. ( **D** ) Three-dimensional reconstruction. Asterisks (*) mark thrombosed pseudoaneurysm. Arrowheads mark vertebral body erosions.

After multidisciplinary agreement, the patient underwent endovascular endoprosthesis deployment at the level of the proximal anastomosis. Due to the asymptomatic status of the focal vertebral erosion lesions, and in the absence of signs of osteoporosis, the patient did not need to undergo any additional vertebral procedures.

The postoperative course was uneventful, with continuous negative blood culture results. MDCT follow-up exams demonstrated exclusion and reduction in size of the anastomotic pseudoaneurysm, with stability of vertebral lytic lesions.

Anastomotic false aneurysm is a rare but possible clinical and anatomical complication after AAA open repair. 1 2 Possible evolution of anastomotic pseudoaneurysm of aortic grafts with vertebral erosion is a very rare event, especially without its infectious contamination. 2 3 4 This situation is only described in few papers in the literature. 4 In patients treated by surgical vascular prosthesis with vertebral erosion onset, the diagnosis of possible anastomotic pseudoaneurysm of aortic grafts should be suspected and evaluated in a differential diagnosis including bone tumors (primary/metastases) and vertebral infections.

In suspicion or confirmation of anastomotic pseudoaneurysm of infectious nature, a surgical revision is the indicated procedure. On the contrary, an endovascular treatment becomes a first-line procedure in noninfected cases. 5 Cases with extensive and unstable vertebral erosion require a second intervention with requisite vertebral column stabilization. 4

In conclusion, in our case we decided to perform an endovascular treatment given the noninfectious nature of the aortic anastomotic pseudoaneurysm with the aim of excluding the pseudoaneurysm sac, with goals of endo-sac pressure reduction, decreased sac volume, and finally prevention of progression in vertebral body erosion.
